# An audit to detect gender-based violence victim-survivors and to improve their referral care pathways in a primary care clinic’s emergency centre in Cape Town, South Africa: A narrative report

**DOI:** 10.4102/safp.v68i1.6280

**Published:** 2026-04-23

**Authors:** Mosedi Namane, Salmaan Moosa

**Affiliations:** 1 Division of Family Medicine, Faculty of Health Sciences, University of Cape Town, Cape Town, South Africa; 2 Vanguard Community Health Centre, Department of Health and Wellness, Cape Town, South Africa

**Keywords:** gender-based violence, audit, primary care, emergency centre, psychosocial referral, medico-legal, Cape Town, South Africa

## Abstract

**Background:**

South Africa’s population experiences high rates of gender-based violence (GBV), with primary care emergency centres (ECs) often being the first point of contact for victim-survivors of violence. The ability of these centres’ staff to identify survivors of abuse plays a critical role in connecting them to a multidisciplinary network of support services. During 2025, a medical intern expressed his frustration with a community health centre’s lack of readily available guides to refer GBV victim-survivors for psychosocial support. Once, it was established that his perception was shared by other clinicians, the family physician of the facility agreed to work with the intern to ‘do a situational analysis audit and fix the problem’.

**Methods:**

The folders of the first 100 adults over 18 years old who presented to the EC with injuries in 2025 were reviewed between June and August for identification of physical and nonphysical abuse plus whether their management included appropriate referral for psychosocial support.

**Results:**

Roughly a quarter were GBV, with most being women. Perpetrators were largely known individuals. For sexual violence, the standard management guidelines were followed, and appropriate medico-psychosocial referrals were made. For non-sexual assaults, such referrals were sparse. Where medico-legal action was taken, it tended to be patient initiated.

**Conclusion:**

These findings pointed to missed opportunities to provide legislated care for abused victims.

**Contribution:**

A flowchart, in line with South Africa’s National Strategic Plan for GBV, was produced to close the gaps. Its generic design will allow for wider use at the primary level.

## Introduction

There are different ways in which gender-based violence (GBV) is defined. The popular definition is the 1993 United Nations general assembly one which states: ‘any act of physical, sexual or psychological harm or suffering to women – including threats of such acts, coercion, or arbitrary liberty deprivation – whether occurring in public or private’.^1^ In our study, we used the South African definition where victims encompass all genders.^2,3^

South Africa has one of the highest rates of GBV.^2^ Primary care emergency centres (ECs) are often the first point of contact for survivors of GBV, resulting in primary care physicians being key in identifying these at-risk individuals to initiate the appropriate management, both in terms of acute medical care and social intervention.^4,5^ Despite SA having strong laws, policies and active civil society,^3,6^ it is ineffective in coordinating government health, police and social services to provide comprehensive support for survivors of GBV and to prevent continuous harm by perpetrators.^4^ The SA GBV framework is multisectoral and is guided by the National Strategic Plan on gender-based violence and femicide (NSP-GBVF) 2020–2030.^3^ It aims not only to respond to violence but also to prevent it through social change, justice reform, survivor support and economic empowerment.

The co-author (SM) and one of the interns who has done a family medicine rotation at Vanguard CHC in 2025, raised an alarm that when he worked in EC, he observed that the needs of victim-survivors of violence could not be sufficiently addressed as there was no guide. This was different from other EC management systems that displayed referral pathways. SM had found it onerous to refer a recent patient seen who required both medico-legal and psychosocial support services. This is what led to the decision to do a comprehensive health-system’s improvement plan to improve the clinic’s service to future GBV victim-survivor clients.

### Aim

To improve both the identification of GBV survivors presenting at a primary care EC in a Cape Town township, South Africa and to enhance the use of referral care pathways for psychosocial and medicolegal support in accordance with the legal framework of SA.^4,6^

## Research methods and design

To gauge the perceived inadequacy for GBV support services, we reviewed the first successive 100 electronic Hospital and Emergency Centre Tracking Information System (HECTIS) and the corresponding paper-based clinical records of adult patients over 18 years old, who presented with a history of assault to Vanguard Community Health Centres’ emergency department 2025. The records were retrospectively audited over June–August for GBV status, documentation of history of physical abuse, documentation of history of emotional abuse, diagnosis according to HECTIS, relationship with assailant, completion of J88 (a medical legal document completed by healthcare professionals that details injuries sustained by alleged victims) and for psychosocial support as per the National and Western Cape’s GBV source documents.^3,7^ No other clinical information was engaged with.

The data were captured in an excel spreadsheet designed by both authors (referred to as MN and SM), sanitised of any patient or staff identifying characteristics and password protected. Data were also only collected from the notes of the index assault-related visit. Where there was recidivism,^5^ the first visit for an assault in 2025 was chosen. There were regular feedback meetings between SM and MN to assess progress and to troubleshoot for concerns like missing data. Permission to do the project was granted by MN and the facility manager of Vanguard CHC.

### Ethical considerations

Permission to share the findings was granted by the Facility manager of Vanguard Community Health Centre, Department of Health and Wellness, Western Cape Government on 09 March 2026.

## Discussion of findings

The first 100 records were identified in the January to June 2025 presentations (Table 1). About a quarter of patients were suspected GBV victims with women disproportionately affected. Where perpetrators were indicated, it was largely known individuals documented for e.g. as intimate partners and co-residents. None of the records had documentation of non-physical forms of abuse such as verbal, emotional, economic or psychological abuse.^8^ The two patients who experienced sexual violence were appropriately referred to the draining Thuthuzela Care Centre (TCC).^6,8,9^ However, the referral rates of patients who were discharged home, to internal and external social and counselling services, were low. Also, processes that required completion of J88s were noted to be largely self-initiated by victims of violence. Collectively, the staff suggested a visual aid be drafted for use in EC to close gaps identified by the audit and to ensure holistic management as per South African legislative framework to support the NSP-GBVF 2020.^3^

**TABLE 1:** Key findings of audit.

Variable	2025						
	January	February	March	April	May	June	Total
	*n*	*n*	*n*	*n*	*n*	*n*	*n*
Patients presenting with assault	8	11	35	34	9	3	100
GBV cases	2	1	4	10	4	2	23
Social services referrals	0	0	0	2	1	0	3
Thuthuzela Care Centre Referrals	0	0	0	1	1	0	2
J88s completed	3	2	5	9	5	1	25
J88s completed by GBV victim-survivors	1	0	1	3	2	0	7

Note: The J88 form is a medico-legal document used in South Africa to document medical examination findings of a victim of crime, like assault or domestic abuse.

GBV, gender-based violence.

**FIGURE 1:**
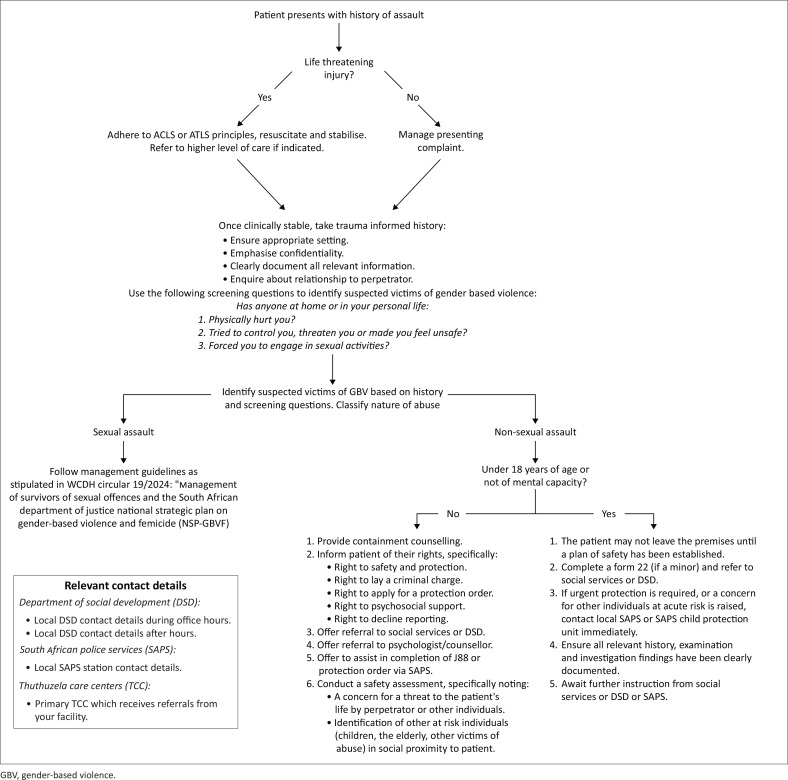
A process for management of suspected victim-survivors of abuse in the emergency centres of Western Cape Department of Health primary healthcare clinics.

The flow chart shown in Figure 1 was designed after a round of meetings. It was deliberately made to be a simple one pager and be generic to allow for cross-use by other primary care clinics.

### Recommendations

The family physician’s (MN) reflection on the project brought the following points to mind:

- The audit was not about getting precise numbers of how the facility was or was not compliant with the law. It was used by the team to introspect, identify shortcomings and take a collective responsibility to correct.
- While doing the project a mentor–mentee relationship developed organically between the family physician and the intern. However, somewhere in the journey a role-reversal occurred with the intern leading the family physician and others in advocacy for social justice.
- A revelation occurred at the initial stages of refining the flowchart that some of the internal and external referral pathways needed to be re-established.
- As clinical head of the facility, it was pleasing to inadvertently experience the improved record system of the facility on ‘a bigger scale’ when 100 patient folders requested could be pulled out over a short period and filed back after the review, seamlessly. This indicated that our facility had been responsive to an identified challenge in our previous local study,^10^ where a major recommendation by our staff had been ‘to campaign for a reliable storage and retrieval of folders’. Our facility has not only gone to attain 1st prize in 2024 for a folder hygiene project in the innovation awards for our substructure, but it also subsequently received a bronze prize for the best primary care team in the Provincial Awards of 2025. We therefore have trust that this newly implemented corrective guide will succeed.

Furthermore, it is recommended to:

- Write a letter to HECTIS management suggesting the inclusion of specific codes for GBV-related physical and nonphysical injuries in HECTIS.
- To have the flowchart be included in the training material for GBV guidelines at the primary care level.
- Repeat the audit in 12 months’ time after the introduction of the flow chart to assess for improvement in psycho-social referrals for GBV victim-survivors.

### Limitations

The internal validity of the audit was reduced by a small sample size, convenient sampling, a single-centre design and a small audit team of two members. Nevertheless, our impression remained that the data still gave us a good idea of what was happening at the study site.

The lack of specific codes of interpersonal violence that could be identified or easily identified on HECTIS. This could be a shortcoming for doctors and nurses to document good-quality medical notes.

## Conclusion

The audit was used to investigate a blind spot that was highlighted to us by a medical intern. Subsequently the findings of the project supported the perception of lack of integration of psychosocial services to support victim-survivors of GBV and other forms of abuse. A visual aid was developed for use in EC to close this gap and to align with the South African legislative framework for NSP-GBVF of 2020. The flowchart produced is generic to allow for use by any local primary care facilities. This is a positive response to our Western Cape Government’s plea (through Circular H19/2024) that is calling for access points for the integrated management of GBV and sexual violence to ‘multiply’.^7^
